# Interactive forces between lignin and cellulase as determined by atomic force microscopy

**DOI:** 10.1186/1754-6834-7-65

**Published:** 2014-04-17

**Authors:** Chengrong Qin, Kimberley Clarke, Kecheng Li

**Affiliations:** 1 College of Light Industry and Food Engineering, Guangxi University, 100 University Road, Nanning, Guangxi Province 530004, PR China; 2 Department of Chemical Engineering, University of New Brunswick, 2 Garland Court, Incutech Complex, Fredericton, NB E3B 5A3, Canada

**Keywords:** Non-productive binding, Lignin, Enzymatic hydrolysis, Atomic force microscopy, Cellulase

## Abstract

**Background:**

Lignin is a complex polymer which inhibits the enzymatic conversion of cellulose to glucose in lignocellulose biomass for biofuel production. Cellulase enzymes irreversibly bind to lignin, deactivating the enzyme and lowering the overall activity of the hydrolyzing reaction solution. Within this study, atomic force microscopy (AFM) is used to compare the adhesion forces between cellulase and lignin with the forces between cellulase and cellulose, and to study the moiety groups involved in binding of cellulase to lignin.

**Results:**

*Trichoderma reesei*, ATCC 26921, a commercial cellulase system, was immobilized onto silicon wafers and used as a substrate to measure forces involved in cellulase non-productive binding to lignin. Attraction forces between cellulase and lignin, and between cellulase and cellulose were compared using kraft lignin- and hydroxypropyl cellulose-coated tips with the immobilized cellulase substrate. The measured adhesion forces between kraft lignin and cellulase were on average 45% higher than forces between hydroxypropyl cellulose and cellulase. Specialized AFM tips with hydrophobic, -OH, and -COOH chemical characteristics were used with immobilized cellulase to represent hydrophobic, H-bonding, and charge-charge interactions, respectively. Forces between hydrophobic tips and cellulase were on average 43% and 13% higher than forces between cellulase with tips exhibiting OH and COOH groups, respectively. A strong attractive force during the AFM tip approach to the immobilized cellulase was observed with the hydrophobic tip.

**Conclusions:**

This work shows that there is a greater overall attraction between kraft lignin and cellulase than between hydroxypropyl cellulose and cellulase, which may have implications during the enzymatic reaction process. Furthermore, hydrophobic interactions appear to be the dominating attraction force in cellulase binding to lignin, while a number of other interactions may establish the irreversible binding.

## Background

Efficient enzymatic hydrolysis of component cellulose to glucose is critical for the production of bioethanol from lignocellulose biomass [1]. Lignocellulose biomass is a complex material consisting of cellulose, hemicellulose, and lignin. The structure of lignocellulose has evolved to protect the cell wall from microbial attack, which is known as biomass recalcitrance. A pretreatment process is often employed prior to the enzymatic hydrolysis reaction to alter the physical and chemical structure of lignocellulose, to increase the cellulose to glucose conversion. Currently pretreatment technology cannot effectively overcome highly recalcitrant material such as forest biomass, in which physical and chemical barriers such as lignin are hard to remove without a significant amount of energy input [1,2].

Lignin is a complex, cross-linked aromatic and hydrophobic polymer consisting of phenylpropane units, that is, guaiacyl propanol, *p*-hydroxyphenyl propanol, and syringyl propanol. The lignin components are then joined together by C-C and aryl-ether linkages with a number of functional groups, including methoxy groups, phenolic hydroxyl groups, and side terminal aldehyde groups [3,4]. Variation is seen within the composition, bonding types, and functional groups of lignin and is ultimately dependent upon the biomass species. This variation will attribute to the recalcitrant characteristic of lignin. Within lignocellulose biomass, lignin is physically and chemically in close association with hemicellulose and cellulose, providing structural support and impermeability to the cell wall, thus creating a physical and chemical barrier [3]. In the enzymatic hydrolysis of the lignocellulose process, lignin will not only physically block cellulase enzymes, but also adsorb them causing a process known as non-productive binding [5].

Previous studies have shown that the addition of lignin to a pure cellulose substrate can reduce sugar release by up to 60% during the enzymatic hydrolysis reaction [6]. Inhibition of the hydrolysis reaction can be attributed to chemical interference, as physical blocking of enzymes by lignin within these experimental conditions is considered insignificant. This inhibition has been attributed to enzyme binding with lignin, which reduces the overall enzyme activity and immobilizes some of the free enzymes so that they are no longer accessible to cellulose [6-8]. The impact of lignin on cellulose degradation was studied by Seawalt *et al*. using cellulosic hydrogels [9]. Untreated hardwood lignin and hydroxypropylated lignin (with blocked lignin phenolic groups) were added to the hydrogel cellulose hydrolysis reaction, to illustrate that the untreated lignin reduced hydrolysis significantly more than hydroxypropylated lignin, thus suggesting that the phenolic groups of lignin mediated the favorable enzyme-lignin interaction. Interactions between lignin-phenolic groups and cellulase enzymes were also found by other researchers [6,10-13], as they attributed the non-productive binding to hydrophobic interactions between cellulase and lignin. Berlin *et al*. [14] also investigated the non-productive binding via nuclear magnetic resonance (NMR) to compare the physical and chemical properties of various lignin preparations and study the inhibition of each substrate. While results obtained by Berlin *et al*. were consistent with the theory that hydrophobic interactions play a major role in non-productive binding, they further suggested that the presence of charged or partially charged functional groups on both lignin and the enzyme surface could also promote lignin-enzyme interactions.

To date, all studies of non-productive binding between lignin and cellulase enzymes have focused on gross adsorption throughout the enzymatic hydrolysis reaction to explain the binding interaction. A direct binding experiment would better elucidate what functional group interactions are involved in non-productive binding and their relative importance.

Atomic force microscopy (AFM) can be utilized to evaluate the interactive forces between cellulase and lignin. Unlike other methods which have been used to study non-productive binding, AFM has the unique ability to directly measure specific force interactions. AFM measures the force between a tip (located on the end of a thin cantilever) and a surface. Interactions between the tip and the surface will cause cantilever deflections, which are recorded on a high-precision optical detector [15,16]. Using Hooke’s law, the magnitude of the interaction force can be determined from the stiffness of a cantilever, *k*, and the cantilever deflection. Furthermore, the AFM probe can be manipulated or designed to have specific chemical or even biochemical characteristics, giving AFM the unique ability to measure force interactions between specified materials and/or molecules.

Previously AFM has been used to study the binding mechanism of the carbohydrate-binding module (CBM) to cellulose [17-19]. Zhang *et al*. [19] developed an AFM functionalized tip which contained a modified CBM unit for single molecule dynamic force spectroscopy (SMDFS). This method studies the specific interactions between a single CBM molecule and cellulose, from which the dynamic and kinetic parameters can be determined. The SMDFS method using a CBM functionalized tip has provided novel information of the mechanism behind cellulase and cellulose interactions. However, this method cannot be used to study the non-productive binding between cellulase and lignin. When non-specific interactions are made between the CBM and substrate, the bridge required for the SMDFS method is not formed, thus the top peak of the oscillation curve is not affected and the interaction is not measured.

The objective of this investigation was to determine the interactive forces between lignin and cellulase in order to better understand the non-productive binding phenomenon. Forces between a relatively large 4.5 μm particle tip and immobilized cellulase are measured with AFM to obtain the adhesion and attraction forces between two substrates. The large particle tip allows for numerous interaction sites between the substrate and cellulase, as cellulase enzymes are estimated to be approximately 5 to 10 nm in size [20-22]. Cellulase enzymes are not immobilized in any specific orientation, as the cellulase enzyme may approach the substrate at any orientation within a cellulase solution.

The utilized enzyme system within this study is a simplification of the enzymatic process to produce glucose from biomass. In reality cellulases are completely free within the system and their location at any moment will be governed by the thermodynamics of the system. This simplification of the lignocellulose reaction is to focus on the fundamental interactions involved in lignin-enzyme binding.

Lignin is added to the hydrolysis reaction of a model cellulose substrate to confirm non-productive binding through observing changes in the rate and quantity of glucose released. AFM is then utilized to study the interactions that occur during non-productive binding between cellulase and lignin. Kraft lignin- and hydroxypropyl cellulose-coated AFM tips are used in conjunction with the immobilized cellulase system to measure the interactive forces between cellulase and lignin, and between cellulase and cellulose. The mechanism of non-productive binding is further studied by measuring non-specific interactions between the immobilized cellulase system and AFM particle tips functionalized with moiety groups. Such tips include hydrophobic polystyrene (PS) tips, PS tips coated with free hydroxyl groups, and PS tips coated with carboxylic groups, in order to compare hydrophobic, H-bonding, and charge-charged interactions, respectively.

## Results and discussion

### Chemical inhibition of lignin during cellulose enzymatic hydrolysis

Kraft lignin was added to the cellulose enzymatic hydrolysis reaction to demonstrate the chemical inhibition of lignin. The ratios of cellulose to lignin were chosen to demonstrate practical lignin content within a biomass sample and how it will affect the chemical inhibition. Untreated lignocellulose substrate will contain a ratio of cellulose to lignin of approximately 2:1, whereas biomass undergoing a pretreatment aimed at removing lignin will generally increase the ratio to somewhere between 4:1 and 10:1, and biomass undergoing an acidic pretreatment to remove hemicellulose will decrease the cellulose to lignin ratio below 2:1.

**Figure 1:**
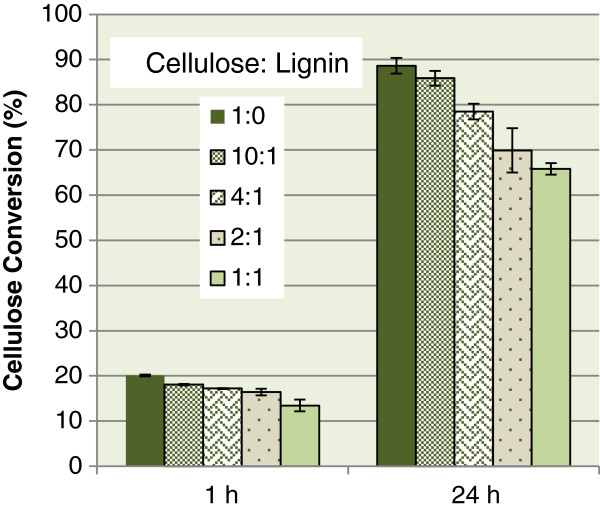
**Cellulose conversion of SBKP substrate throughout enzymatic hydrolysis with varying amounts of Kraft Lignin added to the reaction.** SBKP, softwood bleached kraft pulp.

Figure 1 illustrates the changes in the effectiveness of enzymatic hydrolysis as lignin is added to the hydrolysis reaction. The cellulose to lignin ratio of 1:1 has the greatest inhibitory effect, reducing enzymatic hydrolysis by 33% and 26% after 1 and 24 h, respectively. A 2:1 ratio of cellulose to lignin is shown to decrease glucose yields by 20%, and the reactions with a ratio of 4:1 and 10:1 demonstrated a decrease of approximately 11 to 14% and 3 to 10%, respectively. The trend that the greatest lignin amount will have the least cellulose conversion is as expected, as more lignin will bind with an increase of cellulase enzymes. Lignin inhibition within lignocellulose substrates will differ from these results, as in this study lignin was added to the solution and was not located throughout the lignocellulose structure. Our previous study [23] has shown that untreated softwood substrates (1.7:1 cellulose to lignin ratio) had an enzymatic conversion of 85% lower than a softwood substrate with all lignin removed.

Lignin concentration and type have been shown to similarly affect hydrolysis inhibition [6-8,24]. For instance, Lu *et al*. [8] demonstrated that pretreated softwood with a higher ratio of cellulose to lignin (11:1) was much more digestible (95% conversion) than a low cellulose to lignin ratio substrate (1:1, 45% conversion), and Berlin *et al*. [7] demonstrated that softwood lignin decreased hydrolysis of filter paper by approximately 10%, whereas hardwood lignin decreased the hydrolysis of filter paper by only 2%, with a cellulose to lignin ratio of 7:1.

### Cellulase immobilization

A commercial cellulase system from *Trichoderma reesei*, ATCC 26921, was immobilized onto freshly cleaned silicon (Si) wafers. Si wafers were in 0.10 mg/mL cellulase solution concentrations for varying amounts of time, to determine a suitable time period to produce a relatively even layer of enzymes on the wafer surface. Immobilization of cellulase on the wafer was confirmed with AFM topography imaging (supporting information in Additional file 1: 1 μm × 1 μm three-dimensional AFM topography images of clean Si wafer, *T. reesei*, ATCC 26921, immobilized on a Si wafer for 10 min, 20 min, and 1 h). After 10 min of Si wafer incubation within the cellulase solution there were spaces on the wafer which were not covered with immobilized protein (root mean square (RMS) = 1.4 ± 0.6 nm) and after 1 h of incubation there were proteins over layers (RMS = 4.2 ± 4.8 nm). An incubation time of 20 min of Si wafer within the cellulase solution produced what appeared to be a relatively even layer of protein or enzyme on the Si surface (RMS = 1.3 ± 0.3 nm), thus this incubation time was used for the proceeding laboratory work. It should be noted that the enzymes are within the correct size range of about 5 nm.

Immobilization of a cellulase system from *T. reesei*, ATCC 26921, was executed using a method developed by Tebeka *et al*. [25]. This study varied the incubation time and concentration of the cellulase solution. Tebeka *et al*. immobilized the same cellulase system to determine the adsorption behavior and cellulase activity for the use of immobilized cellulase for enzyme recyclability. The cellulase system immobilized using this technique was shown to retain about 80% of its catalytic activity when compared to free cellulase with the same protein concentration. This suggested that the secondary structure and chemical characteristics of the cellulase proteins are maintained using this technique. The physical adsorption of the cellulolytic cocktail may favor specific enzyme components. However, as a high level of the enzymatic activity was retained it is reasonable to assume that most cellulolytic components have been immobilized, although specific adsorbed cellulase components have not been measured due to the complex procedures it would require.

### Cellulase adhesion to pure lignin and pure cellulose substrate

**Figure 2:**
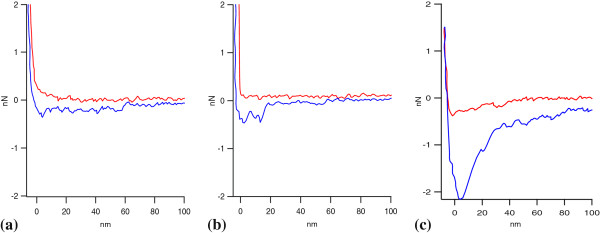
**Representative AFM force curves between the immobilized cellulase system and cellulose-coated tip, lignin-coated tip, and uncoated polystyrene tip. (a)** Cellulose-coated tip, **(b)** lignin-coated tip, and **(c)** uncoated polystyrene tip. AFM, atomic force microscopy.

Lignin- and cellulose-coated tips were used in conjunction with the Si wafer containing the immobilized cellulase via AFM to measure the forces between lignin and cellulase, and between cellulose and cellulase. Representative force curves between hydroxypropyl cellulose and cellulase, and between kraft lignin and cellulase are shown in Figure 2a, b. A representative force curve between an uncoated PS tip and the immobilized Celluclast is also shown in Figure 2c. The differences in the representative force curves observed from the uncoated tip and from both the lignin- and cellulose-coated tips confirm that both the lignin and cellulose were coated on the PS particle. There may be differences in the surface roughness of the coated and uncoated tips, which may account for the difference in the magnitude of the measured adhesion forces. Changing the smoothness of the tip will in turn change the contact area between the tip and surface, which subsequently changes the measured force [26]. Therefore, scanning electron micrograph (SEM) images were obtained (available in Additional file 2), and they indicate that the layer of cellulose and lignin on the tip are relatively even and thin. This warrants an assumption that the lignin- and cellulose-coated particle tips are similar in roughness, so the force values from the two tips are directly compared for different types of interactions.

Force measurements were obtained at room temperature throughout this work. Although enzymatic hydrolysis is normally carried out at 50°C, it has been previously shown that enzymes are still very active within the temperature range of 15 to 50°C with slower kinetics due to lower energy within the system and not changes within the enzyme conformation [27-29]. Therefore, force measurements were obtained at room temperature for easier operation. It should be noted that this may cause minor differences compared with the actual enzymatic hydrolysis condition.

Force curves obtained from cellulose- and lignin-coated tips are very similar upon approach of the cellulase surface. However, upon retraction, adhesion seen between the lignin and the cellulase substrate is significantly larger than that between cellulose and the cellulase substrate. Adhesion forces between the tip and substrate are determined from the force required to pull the tip away from the substrate, after the tip and substrate have made contact. This is illustrated by the maximum negative y-axis values seen within the retraction curve.

**Figure 3:**
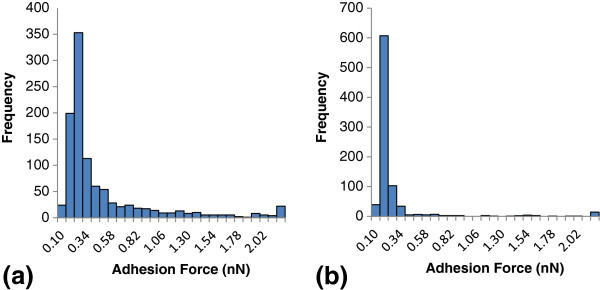
**Histogram of AFM measured forces between the immobilized cellulase system and lignin-coated tip and cellulose-coated tip. (a)** Lignin-coated tip and **(b)** cellulose-coated tip. AFM, atomic force microscopy.

The distribution and frequency of adhesion forces between hydroxypropyl cellulose and immobilized cellulase, and between kraft lignin-coated tips and immobilized cellulase are shown in Figure 3. There were significant differences seen in the measured forces between kraft lignin and cellulase, and those between hydroxypropyl cellulose and cellulase. Forces measured with lignin-coated tips exhibited a force peak at 0.22 nN and the forces measured with the cellulose-coated tips exhibited a peak at 0.16 nN. The average measured adhesion forces between lignin and cellulase (0.45 ± 0.52 nN) was approximately 44% higher than the average adhesion force between cellulose and cellulase (0.25 ± 0.45 nN). A *t*-test was performed on the populations and resulted in a *P* <0.001, indicating a significant difference between the measured adhesion forces with lignin-coated tips and cellulose-coated tips.

Differences in the force interactions of kraft lignin and hydroxypropyl cellulose with cellulase can be attributed to the chemical characteristics of the substrates involved. Lignin is a complex hydrophobic aromatic polymer, with varying side-chains allowing for different chemical interactions, while cellulose is a simple hydrophilic linear polymer, comprised of only glucose molecules. The hydrophilic profile of cellulose has been known to vary due to the strong hydrogen bonds between cellulose chains [30,31]. Furthermore, the cellulose and lignin substrate used within this study are a simplification of the lignocellulose polymers with results that may be different than in lignocellulose enzymatic reaction conditions. The cellulase system used within the system is a commercial product that is commonly used for cellulose hydrolysis. The surface chemical characteristics of immobilized cellulase within the system will vary by the specific cellulase protein. Proteins generally have a hydrophobic core and a hydrophilic surface, which interacts with the aqueous environment they are produced within [32]. However, cellulase databases have shown cellulase enzymes to be relatively rich in surface hydrophobic amino acid residues, thus the surface of most cellulase enzymes appear to have a random and uniform distribution of hydrophobic amino acids [33,34]. Furthermore, the surface of cellulase proteins has been described to be less polar than water. Cellulase surfaces are also rich in hydroxyl groups and carboxylic groups, capable of forming multiple bond types, including hydrogen bonds, charged, and partially charged interactions [33]. The capability of cellulase to form multiple bond types may explain not only the strong attractions between lignin and cellulase, but also the large adhesion forces. Determination of the functional group interactions involved in the non-specific attraction will provide a better understanding of the mechanism of non-productive cellulase binding with lignin.

### Moiety group interactions

**Figure 4:**
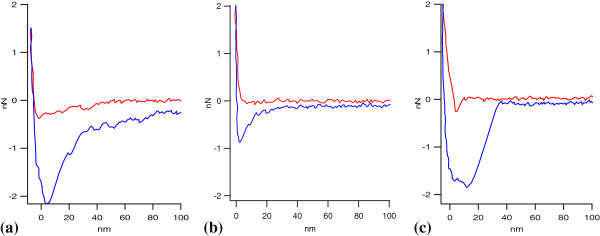
**Representative force curves between the immobilized cellulase system and hydrophobic (polystyrene) tip, chemically modified OH tip, and chemically modified COOH tip. (a)** Hydrophobic (polystyrene) tip, **(b)** chemically modified OH tip, and **(c)** chemically modified COOH tip.

Particle tips with specialized chemistry were used to investigate the force interactions which may contribute to binding of cellulase to lignin. Representative force curves between the immobilized cellulase system and hydrophobic, OH-coated, and COOH-coated tips are presented in Figure 4. Force curves between a PS tip and immobilized cellulase illustrate a strong attraction and adhesion force (Figure 4a). When the approaching hydrophobic tip is about 50 nm from the cellulase, an attractive force pulls the tip onto the immobilized cellulase substrate. As cellulase has been previously discussed to be less polar than water [33], the pull of the hydrophobic tip to the cellulase surface is reasonable, as this change will decrease the overall Gibbs free energy and make the system more thermodynamically favorable. The adhesion force demonstrated as the tip retracts from the surface is about 2.1 nN. Forces between an OH tip and cellulase under the current conditions do not exhibit an attraction force and the adhesion force upon tip retraction is less than 1 nN (Figure 4b). The partially charged COOH tip exhibits a small attractive force at a distance of about 10 nm and the adhesion force upon tip retraction of about 1.8 nN (Figure 4c). The attractive forces are of interest as they may play a role in bringing the enzyme into contact with lignin. Attractive forces between the hydrophobic tip and cellulase are illustrated at a larger distance than between the COOH tip and cellulase.

**Figure 5:**
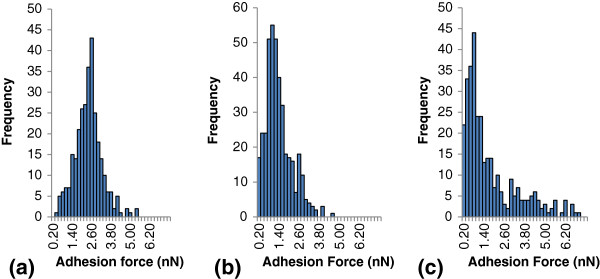
**Histogram of AFM measured adhesion forces between the immobilized cellulase system and hydrophobic (polystyrene) tip, OH chemically modified tip, and COOH chemically modified tip. (a)** Hydrophobic (polystyrene) tip, **(b)** OH chemically modified tip, and **(c)** COOH chemically modified tip. AFM, atomic force microscopy.

The distribution and frequency of adhesion forces between immobilized cellulase and different moiety group tips, including hydrophobic, OH, and COOH tips, are displayed in Figure 5. The corresponding force averages between cellulase and the mentioned moiety groups were 2.3 ± 0.84 nN, 1.3 ± 0.79 nN, and 2.0 ± 2.44 nN, for cellulase interactions with hydrophobic, OH, and COOH tips, respectively. Adhesion forces between the hydrophobic tip and cellulase were 43% and 13% higher than forces measured by OH- and COOH-coated tips, respectively. When comparing the average adhesion forces, hydrophobic-cellulase interactions were the largest. The high variation seen with the COOH tip may be due to the fact that this partially charged group can produce ionic interactions, charge-charge interactions, and H-bonding interactions, which increase force variation. From the histograms, the median adhesion force was determined as the peak on the force distribution histograms with median adhesion forces at 3.0 nN, 2.0 nN, and 0.8 nN, for cellulase interactions with hydrophobic, OH, and COOH tips, respectively. An analysis of variance (ANOVA) was performed to determine if there was any significant difference between the groups, which resulted in *P* <0.001. To directly compare the adhesion forces between the hydrophobic tip and the OH tip, and also between the hydrophobic tip and the COOH tip, directed *t*-tests were performed, in which *P* <0.01 and *P* = 0.10 were obtained, respectively. Thus, there was only a significant difference between measurements obtained with the hydrophobic and OH tip.

All three moiety groups exhibited strong adhesion forces with the cellulase system as measured with AFM. Thus, upon contact of the moiety groups and the cellulase enzymes there were strong interactions between the two substrates. This is reasonable, since where the two substrates are close enough van der Waal forces will become effective as well. The initial attraction force may be of most importance in the process of non-productive binding of cellulase to lignin. These initial forces will bring the cellulase and lignin into contact and then a variety of interactions may keep them adhered together. From the current results, it is seen that hydrophobic interactions have relatively strong and long-range attraction forces to cellulase. Although the other interactions measured do not exhibit a long-range attraction force or attractive force at all under current conditions this does not mean that the attraction force is not present. It does, however, imply that if an attraction force is present, it is not as strong as the hydrophobic-cellulase attraction. This is in agreement with previous results, where the addition of surfactant to the lignocellulose hydrolysis reaction resulted in lignin hydrophobic sites being blocked and with a corresponding decrease in non-productive binding [6,9,10]. This is also in line with the fact that the hydrophobic surface characteristics of cellulase have evolved to attract cellulose [33-35], as cellulose obtained in nature often exhibits hydrophobic characteristics [30].

It should be noted that the cellulase system is complex and the AFM force measurements are tedious. The current result cannot be directly translated to any specific lignocellulose hydrolysis systems. More work is needed within this area to better understand the mechanism of non-productive enzyme binding and to minimize adsorption effects. The interactions of lignin with other cellulase cocktails and single component enzymes should be evaluated, along with varying conditions, such as temperature, pH, and ionic strength, to determine how these variables affect non-productive binding and if there is a consistent mechanism across varying conditions. Different types of lignin preparations should be evaluated as well.

## Conclusions

Kraft lignin was shown to inhibit cellulose enzymatic hydrolysis by up to 30% through enzyme binding. AFM measurements show that within a simplified lignocellulose hydrolysis environment, adhesion forces between kraft lignin tips and cellulase were on average 44% larger than those between hydroxypropyl cellulose tips and cellulase. To understand the mechanism of non-productive enzyme binding, AFM force measurements between the immobilized cellulase and specialized tips which carried specified moiety groups were performed. Interaction forces between hydrophobic tips and cellulase were shown to have the highest adhesion, and were 13% and 43% higher than the average adhesion forces from COOH- and OH-coated tips, respectively. Also, the hydrophobic tips used under the current experimental conditions exhibited attractive forces when the tip was 50 nm away from the immobilized cellulase substrate. The results indicate that within this simplified enzymatic hydrolysis system the hydrophobic-hydrophobic interaction is a major feature in the so-called non-productive binding of cellulase to lignin.

## Methods

### Materials

Softwood bleached kraft pulp (SBKP) was obtained from commercial pulp board. Hydroxypropyl cellulose (average MW = 80,000) and water soluble, low sulfonate kraft lignin (MW = 10,000) was purchased from Sigma-Aldrich (St Louis, MO, USA). Electronic industry grade <100 > and highly polished Si wafer, diced into 5 × 7 mm Si chips, was purchased from SPI Supplies (West Chester, PA, USA).

### Enzymatic hydrolysis

Cellulose enzymatic hydrolysis was carried out using Celluclast 1.5 L and Novozyme 188, with loadings of 15 FPU/g cellulose and 22.5 CBU/g cellulose, respectively. Next, 20 mg/g cellulose of sodium azide was added to the reaction system to prevent bacterial growth. The reaction system had a substrate consistency of 2% (w/w) in 0.05 M sodium citrate buffer (pH 4.8) and was placed within a shaking incubator (model 4450; Thermo Fisher Scientific, Waltham, MA, USA) at 200 rpm and 50°C. Hydrolysis was stopped after a time period of 1 or 24 h. Enzymes were then deactivated by boiling the hydrolysis system for 5 min. Solution from the reaction was separated from the remaining solids via suction filtration. SBKP was used as a model cellulose substrate for enzymatic hydrolysis. Kraft lignin was added at varying concentrations to the enzymatic cellulose hydrolysis reaction.

Cellulose conversion to glucose was determined after the reaction period by measuring filtrate glucose concentration. Filtrate was collected after the reaction and measured for glucose concentration with the YSI 2700 analyzer (YSI, Yellow Springs, OH, USA). From the mass balance, original cellulose content, and amount of glucose produced, the percentage of cellulose conversion to glucose was determined.

### Cellulase immobilization

The cellulase system from *T. reesei*, ATCC 26921 (purchased from Sigma-Aldrich), was immobilized onto Si chips, by a method adapted from Tebeka *et al*. [25]. Si chips were washed, as described elsewhere [36]. Immediately after washing, chips were incubated at 50°C, with 0.10 mg/mL Celluclast concentrations in 0.05 M sodium citrate buffer (adjusted to a pH of 6.5 with a 1.0 M sodium hydroxide solution), for various times. The Si chips were then immediately rinsed well with distilled water and dried overnight within a desiccator. Prior to immobilization the cellulase solution was centrifuged at 3,000 *g* for 10 min, to remove any particles or cell debris within the solution.

### Atomic force microscopy (AFM) probes

Particle PS probes were purchased from Novascan (Ames, IA, USA) for all force measurements within this experiment. All particle tips had a nominal spring constant of 0.06 N/m and a 4.5 μm radius, with a Si nitride cantilever. PS tips with COOH and OH surface chemistry were chemically modified at Novascan, where the tips were coated in gold, then the surface was modified with alkanethiols to produce COOH- and OH-coated probes. Hydroxypropyl cellulose- and kraft lignin-coated tips were produced by dip-coating the PS tips in hydroxypropyl cellulose and Kraft lignin solutions, respectively, as previously described [37].

### AFM force measurements

Forces between substrates were obtained from force-distance curves measured using the MFP-3D™ AFM (Asylum Research, Santa Barbara, CA, USA) in contact mode. All force measurements were carried out at room temperature (approximately 22°C) in 0.05 M sodium citrate buffer (pH 4.8). PS and modified PS tips were used for force measurements with immobilized Celluclast 1.5 L. The actual spring constant of the cantilevers was calculated from the thermal noise of the cantilever with the built-in function of the MPF-3D software. Cantilever sensitivity was recalibrated by pushing the AFM tip against the surface of a glass slide in 0.05 M sodium citrate buffer. A relative force trigger of 0.200 V, a set point of 1.0 V, and a force distance of 1.0 μm were used within all AFM force measurements to reduce measurement variability. As the force trigger, set point, and force distance were consistent among each force measurement and each tip contained the same size radius and spring constant, the obtained forces did not need to be converted to work for direct comparison.

The Si chips containing immobilized cellulase were glued to glass slides with a ceramic adhesive 24 h prior to force measurements. Within each experiment or set of force measurements, a new tip (or modified tip) and freshly immobilized cellulase were used in order to avoid contamination. There were at least 300 force measurements obtained for each measurement type, from three different tips and immobilized cellulase wafers.

### AFM topography imaging

AFM topography images of Si chips and immobilized enzymes on Si chips were obtained using the MFP-3D AFM, in air, non-contact tapping mode. A set point of 500 mV and an integrated gain of 5.0 were chosen for optimizing images. Olympus AC240TS probes (Olympus Corporation, Tokyo, Japan) were used for all topography images. RMS values were calculated using the MFP-3D AFM software from four 1 × 1 μm images for the averages given in the cellulase immobilization section.

## Abbreviations

AFM: Atomic force microscopy; ANOVA: Analysis of variance; CBM: Carbohydrate-binding module; CBU: Cellobiase unit; FPU: Filter paper unit; MW: Molecular weight; NMR: Nuclear magnetic resonance; PS: Polystyrene; RMS: Root mean square; SBKP: Softwood bleached kraft pulp; SEM: Scanning electron micrograph; Si: Silicon; SMDFS: Single molecule dynamic force spectroscopy.

## Competing interests

The authors declare that they have no competing interests.

## Authors’ contributions

CQ was involved in sample preparation. AFM undertook methodology establishment and exploration. KC was involved in the study conception, designed and coordinated the study, drafted the manuscript, and carried out the major laboratory work. KL was involved in the study conception and design, and drafted the manuscript. All authors revised the manuscript, and read and approved the final manuscript.

## Supplementary Material

Additional file 1 **Three-dimensional AFM topography images of 1 μm x 1 μm, with a 50 nm height bar of (a) clean Si wafer (RMS = 0.102 nm), *Trichoderma reesei*, ATCC 26921, immobilized on a Si wafer for (b) 10 min (RMS = 2.08 nm), (c) 20 min (RMS = 1.72 nm), and (d) 1 h (RMS = 2.07 nm). (e)** ANOVA results for RMS values of Si immobilized for varying times. AFM, atomic force microscopy; ANOVA, analysis of variance; RMS, root mean square. Click here for file

Additional file 2 **SEM images of (a, b) cellulose-coated particle tip and (c, d, e) lignin-coated particle tip.** SEM, scanning electron micrograph. Click here for file
