# Supplementary material for: Interactive forces between lignin and cellulase as determined by atomic force microscopy
Source: Biotechnol Biofuels. 2014 Apr 17;7:65. doi: 10.1186/1754-6834-7-65 (PMC4021820; doi:10.1186/1754-6834-7-65)
Supplement: Additional file 1 — Three-dimensional AFM topography images of 1 μm x 1 μm, with a 50 nm height bar of (a) clean Si wafer (RMS = 0.102 nm), Trichoderma reesei, ATCC 26921, immobilized on a Si wafer for (b) 10 min (RMS = 2.08 nm), (c) 20 min (RMS = 1.72 nm), and (d) 1 h (RMS = 2.07 nm). (e) ANOVA results for RMS values of Si immobilized for varying times. AFM, atomic force microscopy; ANOVA, analysis of variance; RMS, root mean square. [file 1754-6834-7-65-S1.pdf]

Supporting Evidence for Cellulase immobilization on Si

(a)

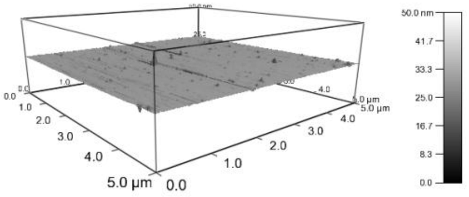

(b)

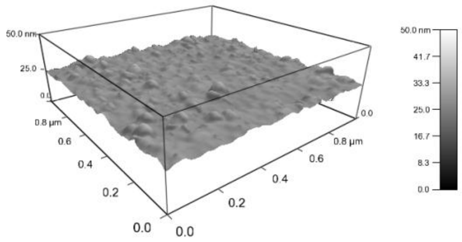

(c)

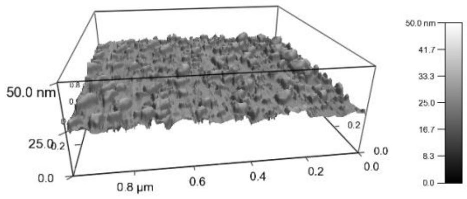

(d)

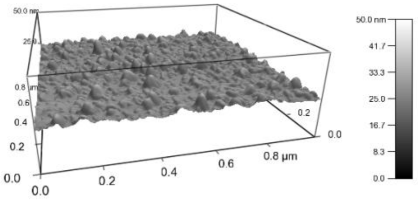

(e)

Anova: Single Factor

SUMMARY

Groups	*Count*	*Sum (nm)*	*Average (nm)*	*Variance (nm)*
Cleaned Si	8	2.42	0.30	0.057
Celluclast 10 min	7	9.75	1.39	0.407
Celluclast 20 min	7	9.21	1.31	0.076
Celluclast 1 h	4	16.9	4.24	23.441

ANOVA

Source of Variation	*SS*	*df*	*MS*	*F*	*P-value*	*F crit*
Between Groups	41.9	3	14.0	4.17	0.017	3.05
Within Groups	73.6	22	3.3			
Total	115.5	25				

Additional File 1. 1µm x 1 µm 3-D AFM topography images, with a 50 nm height bar of (a) clean Si wafer (RMS = 0.102 nm), *Trichoderma reesei*, ATCC 26921 immobilized on a Si wafer for (b) 10 min (RMS = 2.08 nm), (c) 20 min (RMS = 1.72 nm), (d) 1 h (RMS = 2.07nm); (e) ANOVA results for RMS values of Si immobilized for varying time.
