# Supplementary material for: Interactive forces between lignin and cellulase as determined by atomic force microscopy
Source: Biotechnol Biofuels. 2014 Apr 17;7:65. doi: 10.1186/1754-6834-7-65 (PMC4021820; doi:10.1186/1754-6834-7-65)
Supplement: Additional file 2 — SEM images of (a, b) cellulose-coated particle tip and (c, d, e) lignin-coated particle tip. SEM, scanning electron micrograph. [file 1754-6834-7-65-S2.pdf]

Coated PS tips

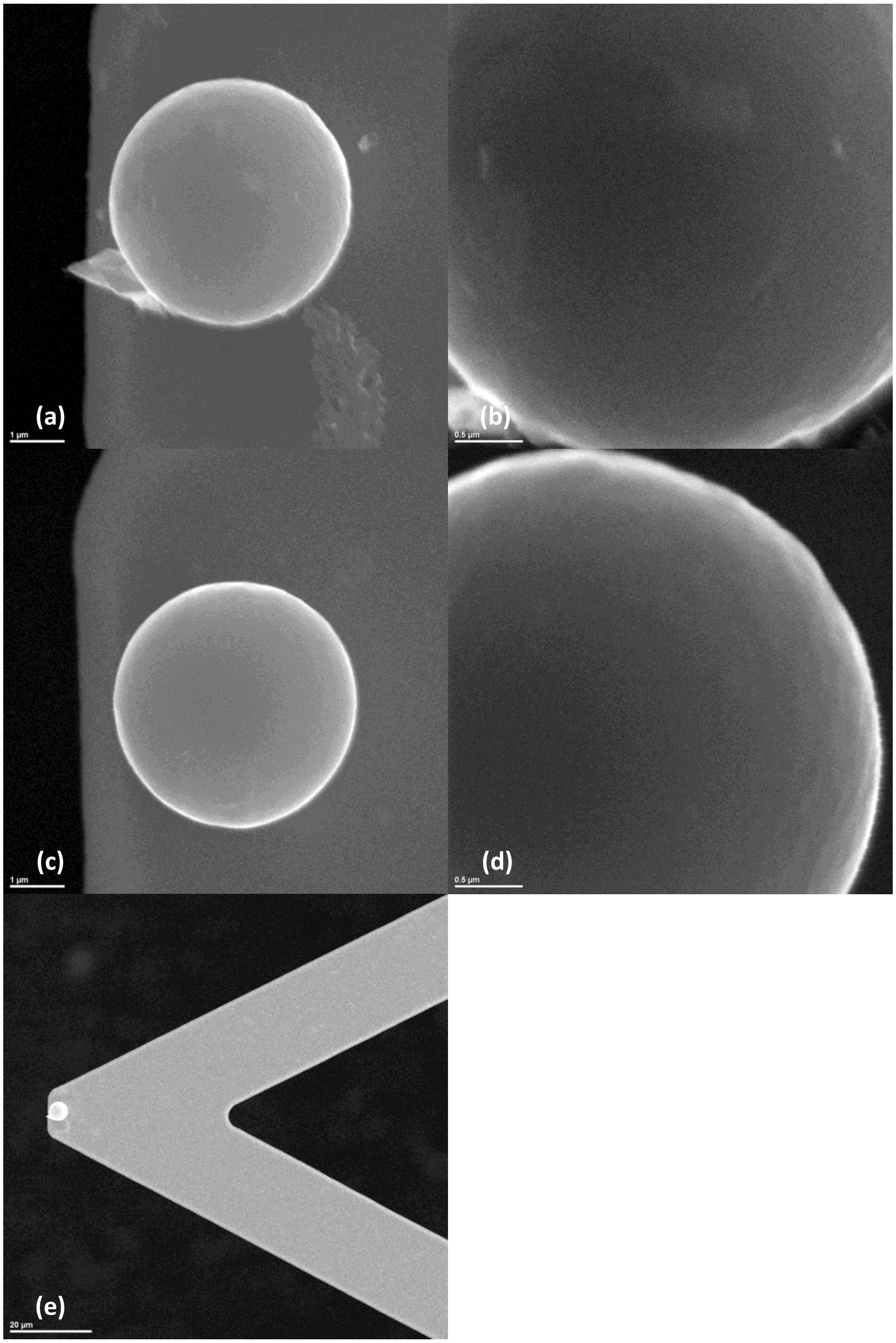

Additional File 2. SEM images of (a, b) cellulose coated particle tip; (c - e) lignin coated particle tip.
